# The usefulness of CYFRA 21–1 to diagnose and predict preeclampsia: a nested case-control study

**DOI:** 10.1186/s12884-016-1132-4

**Published:** 2016-11-03

**Authors:** Lorenz Kuessel, Harald Zeisler, Robin Ristl, Julia Binder, Petra Pateisky, Maximilian Schmid, Julian Marschalek, Thomas Perkmann, Helmuth Haslacher, Heinrich Husslein

**Affiliations:** 1 Department of Gynecology and Obstetrics, Medical University of Vienna, Waehringerguertel 18-20, A-1090 Vienna, Austria; 2 Section for Medical Statistics, Center for Medical Statistics, Informatics, and Intelligent Systems, Medical University of Vienna, Vienna, Austria; 3 Fetal Medicine Unit, St George’s Hospital, Blackshaw Road, London, SW17 0QT UK; 4 Department of Laboratory Medicine, Medical University of Vienna, Vienna, Austria

**Keywords:** Preeclampsia, Diagnosis, Prediction, CYFRA 21–1, Cytokeratin-19, Endothelial dysfunction

## Abstract

**Background:**

The ability to identify patients at risk for developing preeclampsia is important for preventing morbidity and mortality in both the mother and child. Although CYFRA 21–1 (a fragment of Cytokeratin 19) is considered a promising biomarker for diagnosing preeclampsia, little is known regarding the levels of CYFRA 21–1 during pregnancy. Here, we measured serum CYFRA 21–1 levels in women with an uneventful pregnancy and in women whose pregnancy was complicated by preeclampsia. Furthermore we evaluated whether maternal CYFRA 21–1 levels can be used to predict and/or diagnose preeclampsia.

**Methods:**

Longitudinal, sequential blood samples were collected prospectively at seven predetermined visits during pregnancy. Maternal CYFRA 21–1 levels were measured in 50 women with an uneventful pregnancy (control group) and in 10 asymptomatic women whose pregnancy was later complicated by preeclampsia (PE_long group). In addition, CYFRA 21–1 levels were measured from a single sample collected from a separate group of 50 pregnant women with symptomatic preeclampsia (PE_state group).

**Results:**

The CYFRA 21–1 levels were significantly higher in the PE_state group compared to the control group (*p* < 0.001). In the PE_long group, CYFRA 21–1 levels were lower from gestational week 11 through 17, but were higher than the control group from gestational weeks 18 through 36.

Out of the ROC curves that were calculated to investigate the predictive and diagnostic properties of CYFRA 21–1 levels for preeclampsia, the ROC curve for diagnosing preeclampsia in gestational week 28–32 showed the largest AUC of 0.92, at a cut-off point of 3.1 ng/ml, leading to sensitivity of 92 % and specificity of 80 %.

**Conclusions:**

The elevated serum levels of CYFRA 21–1 observed in both groups of women with preeclampsia may reflect endothelial damage and/or dysfunction. Our results suggest that maternal serum CYFRA 21–1 is a promising biomarker for diagnosing preeclampsia. Although its value for predicting the long-term occurrence of subsequent preeclampsia may be limited, our findings indicate a trend towards elevated maternal CYFRA 21–1 levels preceding the short-term occurrence of preeclampsia in asymptomatic women. Additional prospective longitudinal studies are needed in order to determine the value of measuring maternal serum CYFRA 21–1 in predicting preeclampsia.

## Background

Preeclampsia (PE) is a leading cause of perinatal maternal and neonatal morbidity and mortality, affecting 2–8 % of pregnancies in the industrialized world [1, 2]. Although PE places high burden on both the mother and the newborn infant, and despite considerable research over the past few decades, the precise cause and pathophysiology of PE remain largely unknown. The current leading hypothesis is that a defect in early placentation combines with impaired trophoblast differentiation, leading to multi-stage pathogenesis [3, 4].

The initial stage of the disease, which is largely asymptomatic, involves abnormal placentation, which leads to placental ischemia. This placental stage of PE is followed by widespread endothelial damage and dysfunction (known as the maternal stage) mediated by soluble factors from the placenta entering the maternal circulation. Given the high complexity of the disease, screening for PE and establishing a clear diagnosis of PE remain challenging in clinical practice. Nevertheless, the ability to identify patients at risk for PE and the ability to accurately diagnose patients with PE are essential in order to prevent PE-associated morbidity and mortality.

Cytokeratin 19 is a member of the keratin family of intermediate filament proteins responsible for maintaining the structural integrity of epithelial cells [5]. Cytokeratin 19 and other keratins are often used to differentiate cells of epithelial origin from hematopoietic cells in tests that evaluate circulating tumor cells in peripheral blood or trophoblastic particles in maternal circulation [6, 7]. When they occur in the peripheral circulation, cytokeratins are present primarily as partially degraded, individual protein fragments or complexes. Cytokeratin fragment 21–1 (CYFRA 21–1), a fragment produced from Cytokeratin 19, has been studied in patients with lung, esophageal, and gynecological cancers and has been shown to be a clinically useful prognostic marker [8–13]. Cytokeratins and their fragments are released into the circulation via several mechanisms, including cellular apoptosis, necrosis, abnormal mitosis, and/or spillover from proliferating cells, and these cellular mechanisms are also present in patients with PE [14–17].

Our group and others have reported increased levels of CYFRA 21–1 in the serum of preeclamptic women as well as in umbilical cord blood harvested from preeclamptic women [18–21]. Furthermore, serum CYFRA 21–1 levels have associated with disease severity and appear to be unaffected by antihypertensive medications [19, 20]. However, little is known regarding the dynamics of CYFRA 21–1 during pregnancy, regardless of whether the pregnancy is uneventful or complicated by PE. Given that the placental stage of PE precedes the maternal stage, we hypothesized that the pathogenic mechanisms of PE lead to increased levels of CYFRA 21–1, and this increase may precede the onset of PE-associated symptoms, thereby providing a marker that could be used to predict the risk of developing PE and/or to diagnose PE at an early stage.

To test this hypothesis, we measured CYFRA 21–1 levels in serum collected from women who had an uneventful pregnancy and from women who subsequently developed PE. Lastly, we examined the value of using CYFRA 21–1 to predict and/or diagnose PE.

## Methods

### Study design

For this single-center, nested case-control study, biological samples and medical data were obtained from the Biobank for Pregnancies at the Department of Obstetrics and Feto-maternal Medicine, Medical University of Vienna, Vienna General Hospital. This Biobank consists of an electronic database of clinical, sonographic, demographic, and pregnancy outcome data, as well as maternal serum, plasma, and urine samples. The presence of infectious disease and maternal age under 18 years are the only criteria for exclusion from the Biobank. At the time of inclusion, written informed consent is obtained, and each participant is interviewed in order to obtain a detailed patient medical history. The design of the Biobank includes a longitudinal arm and a state-of-disease arm. For the longitudinal arm, all patients who are receiving prenatal care at our department are invited to participate. During their pregnancy, the participants are seen in gestational week 11–13 (visit 1), gestational week 14–17 (visit 2), gestational week 18–22 (visit 3), gestational week 23–27 (visit 4), gestational week 28–32 (visit 5), gestational week 33–36 (visit 6), and after gestational week 37 (visit 7). Each visit includes a medical history assessment, an ultrasound examination, blood sample collection, and a physical examination. After delivery, a final outcome regarding the relevant diagnosis is established for each pregnancy (e.g.,. PE is defined as the final outcome if the participant experienced PE).

In the state-of-disease arm, the participant’s blood samples and data are collected only once (when the pregnancy-related complication is first diagnosed). The participants in the state-of-disease arm are women with an established diagnosis of a pregnancy-related complication who were not included in the longitudinal arm of the Biobank (e.g., patients who received prenatal care at another hospital and were transferred to our hospital due to a suspicion of PE).

### Study groups

After we excluded participants in the Biobank who presented with malignancy, multiple pregnancy, preexisting hypertension and/or proteinuria, chronic renal disease, type 1 or type 2 diabetes, Crohn’s disease, ulcerative colitis, and/or autoimmune disease, the remaining participants were used to create three study groups. The first group included women participating in the longitudinal arm with the final outcome of an uneventful pregnancy (the control group). The second group included women in the longitudinal arm with the final outcome of PE (PE_long). The third group included women with preeclampsia in the state-of-disease arm (PE_state). For the control group, longitudinal serum samples were collected at random from 50 participants with an uneventful pregnancy; we excluded participants with fewer than four of the seven Biobank visits. Pregnancy was defined as uneventful if the participant was normotensive, normoglycemic, had no signs of proteinuria, and delivered a term infant (i.e., at 37 + 0 weeks of gestation or later) that was appropriate size for the gestational age. For the PE_long group, all eligible pregnancies from the longitudinal arm of the Biobank with the final outcome of PE were included (*n* = 10 participants). It is important to note that in this group, samples were obtained only before the participant developed PE; as soon as PE was diagnosed, delivery was initiated and further blood samples could not be collected. Only one post-diagnosis sample was obtained from one participant in this group; this sample was excluded from analysis. Therefore, in order to analyze samples from women with PE, we created a study group consisting of samples obtained from symptomatic women with PE; for this group, 50 cases of PE were selected at random from the state-of-disease arm.

The diagnosis of PE was based on international guidelines [22, 23]. The diagnostic criteria for PE included a new onset of both hypertension (systolic blood pressure ≥140 mmHg and/or diastolic blood pressure ≥90 mmHg) and proteinuria (≥300 mg of protein per 24-h urine collection, or protein to creatinine ratio ≥30 mg per millimole) after gestational week 20; Severe PE was defined by the presence of sustained severe hypertension (two or more recordings of systolic pressure of ≥170 mmHg or diastolic pressure of ≥110 mmHg), or evidence of multisystem disorder; Early-onset PE was defined as PE presenting before 34 + 0 weeks of gestation.

### Sample analysis

Blood samples were collected immediately following a blood pressure measurement and were separated by centrifugation within two hours of collection. Serum samples were stored in aliquots below −70 °C until analysis in order to avoid interference due to repeated freeze-thaw cycles. Serum CYFRA 21–1 was measured using an electrochemiluminescence immunoassay (certified IVD kit, Roche Diagnostics GmbH, Mannheim, Germany) with a cobas e 620 module in a cobas 8000 modular analyzer series (Roche Diagnostics GmbH). Serum (20 μl) was incubated with two specific monoclonal antibodies (KS 19.1 and BM 19.21) against Cytokeratin 19; these two antibodies form a “sandwich” complex with Cytokeratin 19. This complex adheres to an electrode within the measurement cell via a chemical biotin-streptavidin interaction with magnetic microparticles. When voltage is applied, a chemiluminescent signal is emitted from one of the two antibodies, which is labeled with a Ruthenium complex; this signal is then used to calculate the concentration of CYFRA 21–1 using a standard reference curve. The intra-assay coefficient of correlation was 7.1 % at a concentration of 3 ng/ml. All samples were analyzed at the Department of Laboratory Medicine, Medical University of Vienna, which operates a certified (ISO 9001:2008) and accredited (ISO 15189:2008) quality management system.

### Statistical analysis

The time course of CYFRA 21–1 in the control group was visualized graphically for each participant. For further visualization, the median CYFRA 21–1 level measured over time was estimated using locally weighted median regression. Box plots were used to compare the distribution of CYFRA 21–1 levels between the study groups at each visit. To formally test our primary null hypothesis (i.e., equal mean CYFRA 21–1 levels in the control and PE_long groups at each of the first six visits), a linear mixed model was fit and an F-test was calculated for the corresponding comparison. In the model, the mean CYFRA 21–1 level is explained using a distinct combination of treatment group and visit, as well as a random intercept term for each participant, which accounts for the correlation of repeated observations within each participant. Additionally, the same models were fit including maternal age and BMI as co-variables to account for their potential influence on CYFRA 21–1 levels. The differences in CYFRA 21–1 among groups were again tested by F-tests. For this adjusted model, the test aims to provide a comparison of mean CYFRA 21–1 levels between subjects of different groups, though with the same age and BMI. The calculations were performed using the MIXED Procedure in SAS, with the Kenward-Roger method for calculating standard errors and degrees of freedom. A similar test was performed for the analogous secondary null hypothesis (i.e., no difference between the control and PE_state groups at visits 4, 5, and 6). Visit 7 was not included in these analyses, because it was reached only in few pregnancies, rendering information from this visit non-representative for the main analysis goal. As a next step, receiver operating characteristics (ROC) analysis was used to examine the value of using CYFRA 21–1 level to predict the risk of PE. For each possible predictor, we calculated the area under the ROC curve (AUC) and the 95 % confidence interval.

For analyzing demographic data and secondary outcomes, mean and standard deviation, or median and range were calculated for metric variables and absolute and relative frequencies were calculated for categorical variables. Secondary outcomes were compared between groups using an analysis of variance (ANOVA), Student’s *t*-test, or Fisher’s exact test, where appropriate.

## Results

### Characteristics of the study groups

The demographic data and pregnancy outcome results of the three groups are summarized in Table 1. No significant difference was observed between the groups with respect to gestational age at each visit (Table 2).

**Table 1:** Patient characteristics and pregnancy outcome parameters

	Total	Control (1)	PE_long (2)	PE_state (3)	p global	p 1 vs 2	p 1 vs 3
n	110	50	10	50			
Maternal Age	31.28 ± 6.4	29.9 ± 6	32.1 ± 6.4	32.5 ± 6.63	0,116^a^	0,336^b^	0,042^b^
Maternal BMI	24.99 ± 4.8	23.44 ± 3.9	27.2 ± 3.53	26.09 ± 5.38	0,006^a^	0,009^b^	0,006^b^
Smoking in pregnancy					0,057^c^	0,715^c^	0,040^c^
NO	89 (81 %)	36 (72 %)	8 (80 %)	45 (90 %)			
YES	21 (19 %)	14 (28 %)	2 (20 %)	5 (10 %)			
Parity					0,002^c^	0,406^c^	<0.001^c^
0	50 (48 %)	19 (38 %)	4 (40 %)	27 (61 %)			
1/2	46 (44 %)	30 (60 %)	5 (50 %)	11 (25 %)			
> 2	8 (8 %)	1 (2 %)	1 (10 %)	6 (14 %)			
GA at Delivery (weeks)	35.75 ± 4.84	39.59 ± 1.27	34.97 ± 4.42	32.05 ± 4.23	<0.001^a^	0,009^b^	<0.001^b^
Mode of Delivery					<0.001^c^	<0.001^c^	<0.001^c^
Caesarean section	70 (64 %)	17 (34 %)	10 (100 %)	43 (86 %)			
Vaginal delivery	40 (36 %)	33 (66 %)	0 (0 %)	7 (14 %)			
Neonatal birth weight (grams)	2471.23 ± 1132.82	3403.8 ± 398.16	1988.2 ± 784.89	1635.26 ± 966.85	<0.001^a^	<0.001^b^	<0.001^b^
Transfer of newborn to NICU					<0.001^c^	<0.001^c^	<0.001^c^
NO	72 (65 %)	50 (100 %)	4 (40 %)	18 (36 %)			
YES	38 (35 %)	0 (0 %)	6 (60 %)	32 (64 %)			
Gender of newborn					0,324^c^	0,299^c^	0,840^c^
female	59 (54 %)	27 (54 %)	3 (30 %)	29 (58 %)			
male	51 (46 %)	23 (46 %)	7 (70 %)	21 (42 %)			

Categorical data are presented as the frequency and percentage (rounded). Continuous variables are expressed as the mean ± SD

*GA* gestational age, *BMI* pre-prenancy body mass index, *NICU* neonatal intensive care unit

^a^ANOVA; ^b^Student’s *t*-test; ^c^Fisher’s exact test

**Table 2:** Gestational age and CYFRA 21–1 serum levels at the seven sample collection time points during pregnancy

visit		Control	PE_long	PE_state	Total
1	n	50	8	0	58
	GA	12.14 (11.75–12.68)	12.43 (12.32–13.04)		12.14 (11.86–12.71)
	CYFRA	2.35 (2.1–2.77)	1.75 (1.48–2.15)		2.3 (1.9–2.7)
2	n	47	9	0	56
	GA	16.14 (15.36–16.79)	16.57 (16.14–17)		16.29 (15.43–16.86)
	CYFRA	2.2 (1.8–2.6)	2.1 (1.8–2.6)		2.15 (1.8–2.6)
3	n	50	9	1	60
	GA	20.57 (20.32–21)	20.57 (20.14–20.86)	22.86 (22.86–22.86)	20.57 (20.25–21)
	CYFRA	1.9 (1.52–2.4)	2 (1.7–2.5)	3 (3–3)	1.9 (1.58–2.42)
4	n	47	8	12	67
	GA	25.14 (24.71–25.93)	24.79 (24.21–25.36)	25.5 (24.96–26.93)	25 (24.71–26.07)
	CYFRA	2 (1.65–2.55)	2.35 (2.18–3.43)	3.8 (2.82–4.12)	2.3 (1.85–2.9)
5	n	45	7	13	65
	GA	30 (28.86–31.14)	29 (28.93–30.71)	30.14 (28.57–31.71)	30 (28.71–31.29)
	CYFRA	2.3 (1.9–2.9)	2.6 (2.45–3.4)	4.7 (3.9–5.5)	2.6 (2.1–3.9)
6	n	47	6	19	72
	GA	34 (33.29–35.29)	33.36 (33.07–33.64)	34.86 (33.71–35.5)	34 (33.39–35.43)
	CYFRA	3.2 (2.5–3.8)	3.8 (3.3–5.12)	6 (4.55–6.85)	3.65 (2.85–5.58)
7	n	31	1	5	37
	GA	37.57 (37–38.14)	37.86 (37.86–37.86)	37.29 (37–37.29)	37.43 (37–38.14)
	CYFRA	3.7 (2.9–4.35)	4.3 (4.3–4.3)	4 (3.7–4.5)	3.8 (3–4.5)

GA and CYFRA values are presented as the median (interquartile range)

*GA* gestational age in weeks, *CYFRA* serum CYFRA 21–1 levels in ng/ml

### CYFRA 21–1 levels in the control group

**Fig. 1:**
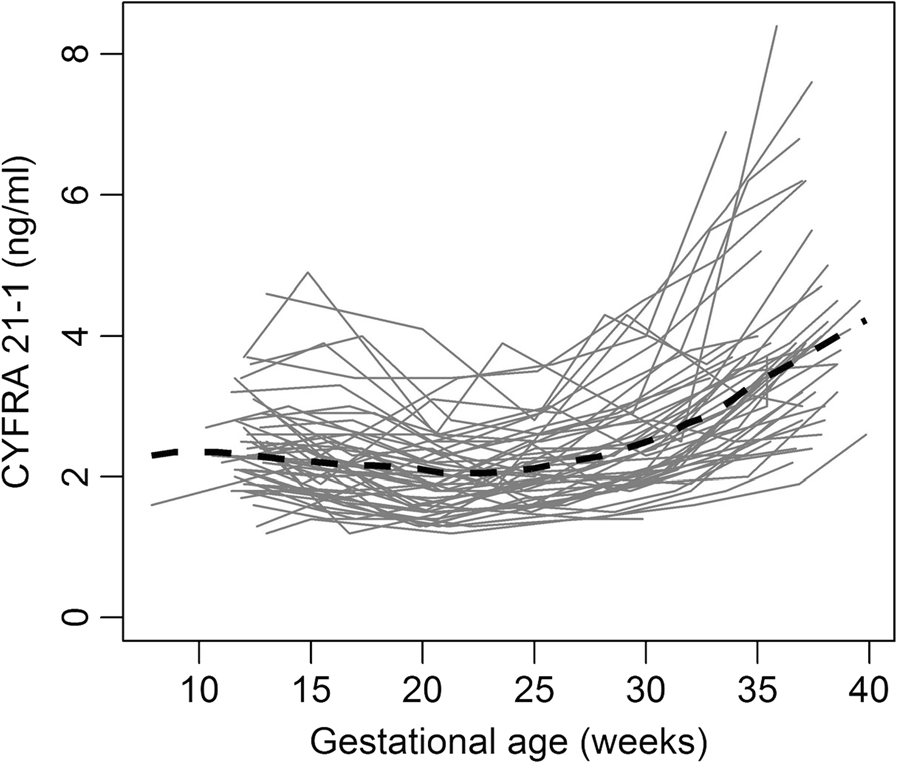
CYFRA 21–1 over time in uneventful pregnancies. Serum CYFRA 21–1 concentration as a function of gestational age in the control group of women with an uneventful pregnancy

First, we measured serum CYFRA 21–1 levels in the women who had an uneventful pregnancy (control group) in order to examine the relationship between gestational age and serum levels. The individual and median serum values of CYFRA 21–1 in the control group are shown in Fig. 1.

### CYFRA 21–1 levels in the PE_long group

Next, we measured the serum CYFRA 21–1 levels in the PE_long group, which contains asymptomatic pregnant women who developed PE later in their pregnancy. We then performed an F-test to investigate the global difference between the control and PE_long groups using a mixed model that explains the mean serum levels of CYFRA 21–1 across pregnancy, yielding a *p*-value of 0.080. With this analysis, the null hypothesis of equal development of mean CYFRA 21–1 levels in both groups could not be formally rejected at the 5 % significance level.

As women in the PE_long group were significantly older and had higher pre-pregnancy BMIs compared to controls, we decided to investigate the influence of these characteristics on CYFRA 21–1 levels and to calculate another mixed model, which additionally included the maternal variables age and BMI. In this adjusted model, the effect of BMI led to an estimated average reduction of CYFRA 21–1 by 0.05 (SE 0.024) ng/ml for each unit increase in BMI; The influence of the co-variable age was estimated with average reduction of 0.015 (SE 0.015) ng/ml per one year increase in age. The analysis of the adjusted model yielded a significant global difference between the control and PE_long groups (*p* = 0.023).

**Fig. 2:**
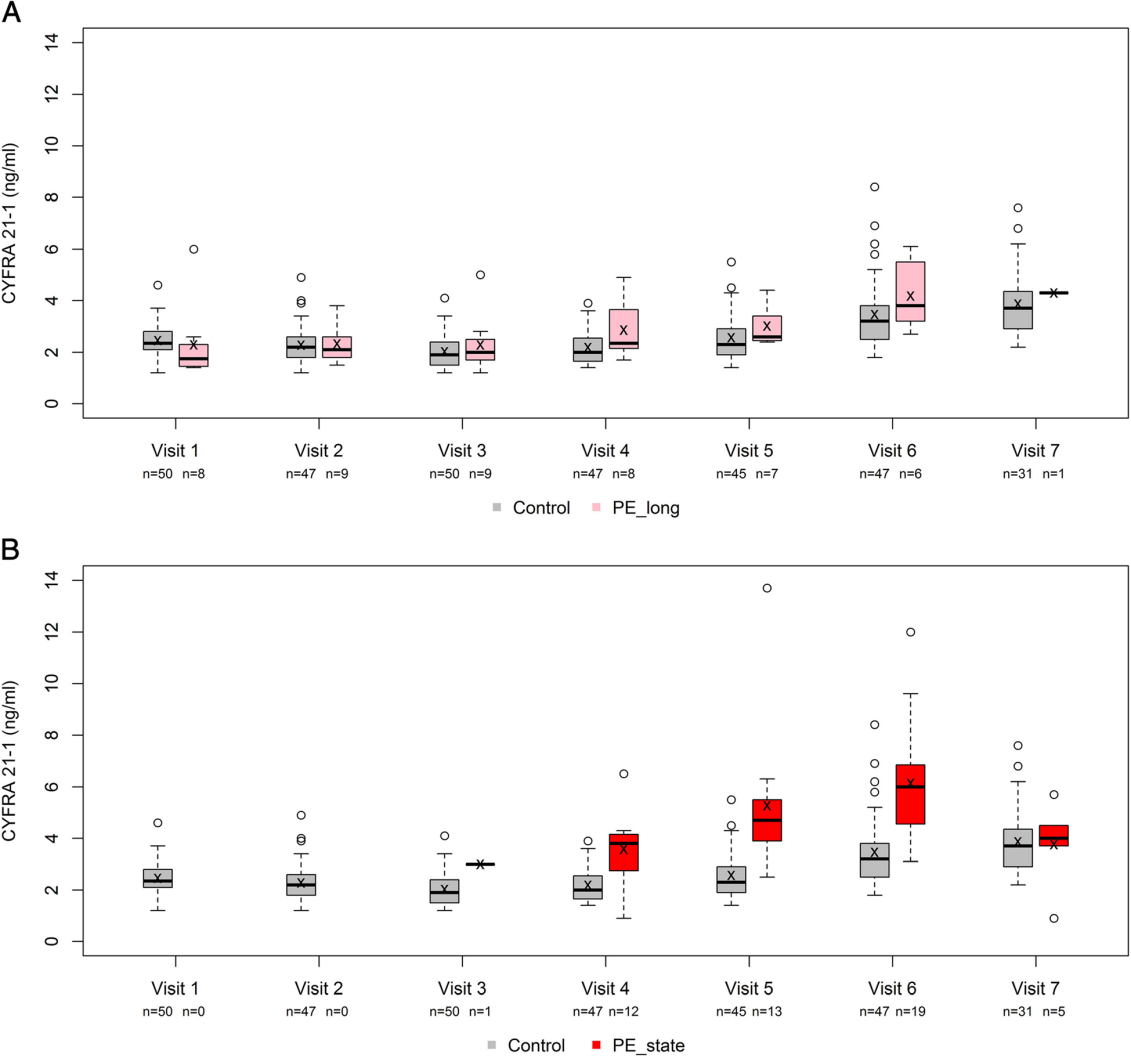
CYFRA 21–1 levels in the study groups. The median CYFRA 21–1 levels at each visit are shown in the control group and in the PE_long (**a**) and PE_state (**b**) groups

The median serum levels of CYFRA 21–1 during the pregnancy are shown in Fig. 2a. At visit 1, the median serum CYFRA 21–1 concentration in the PE_long group and control group was 1.75 ng/ml (range: 1.48–2.15 ng/ml) and 2.35 ng/ml (range: 2.1–2.77 ng/ml), respectively (*p* = 0.046, analyzed using the Kruskal-Wallis test). At visit 2, CYFRA 21–1 concentration was still lower in the PE_long group than in the control group; however, at visits 3, 4, 5, and 6 the levels were higher—albeit not to the level of statistical significance—in the PE_long group than in the control group.

### CYFRA 21–1 levels in the PE_state group

Lastly, we compared serum CYFRA 21–1 levels between the symptomatic women with PE (PE_state) and the control group (Fig. 2b). Our analysis revealed that the serum CYFRA 21–1 levels were higher in the PE_state group compared to the control group (*p* < 0.001 for the F-tests from both the unadjusted and the adjusted models). In the adjusted model, the estimated effect of BMI led to an average CYFRA 21–1 reduction of 0.034 (SE 0.034) ng/ml per unit increase in BMI; one year increase in maternal age led to an average reduction of 0.050 (SE 0.025) ng/ml.

To examine possible differences in CYFRA 21–1 levels between subtypes of PE, the PE_state group (*n* = 50) was split into the subgroups: (i) early-onset PE (*n* = 36 (72 %), median 4.3 ng/ml, IQR: 3.7–5.7) and late-onset PE (*n* = 14 (28 %), median 5.4 ng/ml, IQR: 3.9–6.8) (ii) severe PE (*n* = 43 (86 %), median 4.5 ng/ml, IQR: 3.7–6.2) and non-severe PE (*n* = 7 (14 %), median 4.5 ng/ml, IQR: 3.9–5.0), and (iii) PE cases associated with (*n* = 23 (46 %), median 5.1 ng/ml, IQR: 3.7–6.5) or without (*n* = 27 (54 %), median 4.1 ng/ml, IQR: 3.8–5.6) fetal growth restriction. Subgroup analysis revealed no significant differences among groups (Wilcoxon test, *p* = 0.206, *p* = 0.585, and *p* = 0.213, respectively).

### Diagnosis of PE by measuring serum CYFRA 21–1 concentration

**Fig. 3:**
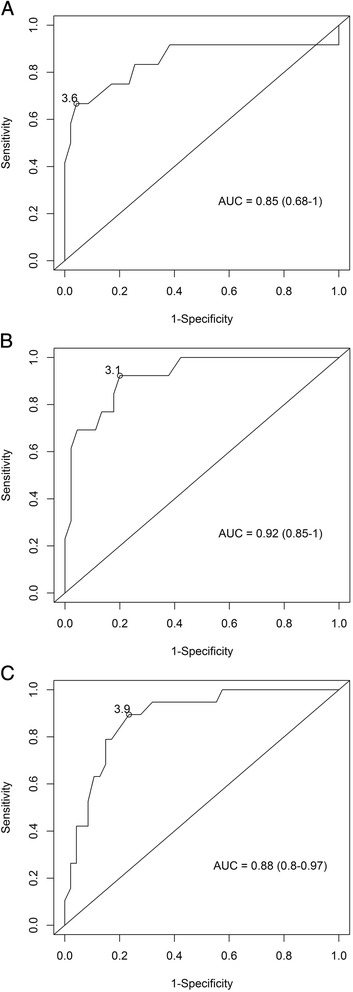
ROC curves. ROC curves showing the sensitivity and specificity of using serum CYFRA 21–1 concentration measured in gestational week 23–27 (**a**), 28–32 (**b**), or 33–36 (**c**) to preeclampsia

Next, we analyzed the predictive value of measuring CYFRA 21–1 levels in diagnosing PE by analyzing the ROC curves from visits 4, 5, and 6 (Fig. 3). The AUC values, 95 % confidence intervals, and cut-off values with the largest sum of sensitivity and specificity are shown in Table 3.

**Table 3:** Predictive power of maternal serum CYFRA 21–1 levels in symptomatic women with preeclampsia (PE_state) and in asymptomatic women who later developed preeclampsia (PE_long)

Predictor	AUC	95 % CI	Cut-off (ng/ml)	Sensitivity	Specificity	*p*-value
For existing Preeclampsia (PE_state)						
CYFRA 21–1 level at visit:						
4	0.85	0.68–1	3.6	0.67	0.96	<0.001
5	0.92	0.85–1	3.1	0.92	0.8	<0.001
6	0.88	0.8–0.97	3.9	0.89	0.77	<0.001
For subsequent Preeclampsia (PE_long)						
CYFRA 21–1 level at visit:						
1	0.28	0.02–0.53	ND^a^	ND	ND	0.088
2	0.51	0.29–0.74	2.5	0.44	0.7	0.909
3	0.54	0.3–0.77	2.8	0.22	0.9	0.785
4	0.71	0.51–0.9	2.1	0.88	0.53	0.037
5	0.70	0.55–0.86	2.4	1	0.51	0.012
6	0.69	0.48–0.9	2.7	1	0.34	0.074
CYFRA 21–1 increase between visits:						
1 and 2	0.46	0.24–0.68	ND	ND	ND	0.724
2 and 3	0.48	0.25–0.71	ND	ND	ND	0.857
3 and 4	0.61	0.41–0.81	0.6	0.67	0.94	0.287
4 and 5	0.66	0.51–0.82	0.6	0.83	0.76	0.042
5 and 6	0.69	0.48–0.9	2.0	0.67	0.85	0.074

^a^Cut-off values, sensitivity, and specificity are not provided if the AUC is <0.5

*AUC* area under the curve, *95 % CI* 95 % confidence interval, *CYFRA 21–1* Cytokeratin fragment 21–1, *PE* preeclampsia

The results of this analysis show, that CYFRA 21–1 levels provide a clear distinction between women with PE and unaffected pregnant controls.

### Using maternal serum CYFRA 21–1 to predict subsequent PE

Lastly, we used ROC AUC analyses to examine whether maternal serum CYFRA 21–1 levels measured at each visit and/or the change in CYFRA 21–1 levels between two visits can be used to predict the subsequent occurrence of PE in asymptomatic woman. These results are summarized in Table 3. The analyzed AUCs, together with the graphical representation in Fig. 2, suggest that CYFRA 21–1 levels at visits 4 to 6 have the potential to provide some distinction between women who will develop PE later in their pregnancy and women with an uneventful pregnancy.

Applying the cut-off values of visit 4, 5, and 6 to our cohort, women in the PE_long group are detected before the clinical diagnosis based on symptoms of PE with a median time interval of 9.2 weeks between prediction and clinical diagnosis; However, due to the limited sample size in the PE_long group, and due to the lack of validation of our findings by an external sample or by cross-validation, this should not be over-interpreted.

## Discussion

The primary goal of this study was to measure the dynamics of maternal serum CYFRA 21–1 levels during uneventful pregnancy and during pregnancy complicated by PE. Our secondary goal was to determine the value of using maternal serum CYFRA 21–1 levels to diagnose PE and/or predict the risk of developing PE in asymptomatic women. Using samples obtained from the prospective longitudinal Biobank study, which includes sequential blood samples obtained at up to seven predetermined time points during pregnancy, we measured the time course of maternal serum CYFRA 21–1 levels. Our results revealed three major findings. First, the time course of maternal CYFRA 21–1 levels differs between uneventful pregnancies and pregnancies that are complicated by PE. Second, serum levels of CYFRA 21–1 are significantly higher in symptomatic women with PE (i.e., in our PE_state group) compared to women with an uneventful pregnancy, indicating that this circulating protein fragment is a promising biomarker for diagnosing PE. Lastly, the value of using maternal CYFRA 21–1 to predict the long-term occurrence of preeclampsia appears to be limited. However, our findings indicate a trend towards elevated maternal CYFRA 21–1 levels preceding the short-term occurrence of preeclampsia in asymptomatic women.

Worldwide, preeclampsia is estimated to account for approximately 50,000 cases of perinatal maternal death each year [2, 24]. Therefore, developing a reliable tool for identifying patients who are at risk for developing PE is an important first step towards preventing PE and PE-associated morbidity and mortality. However, the diverse clinical presentation of PE and the lack of a simple screen have hampered previous attempts to develop such a tool. Recently, biomarkers such as soluble fms-like tyrosine kinase 1 and placental growth factor have been used to diagnose and/or predict the short-term risk of PE in women with a clinical suspicion of PE [25–27]. However, the ability to identify women with a long-term risk for developing later in their pregnancy would constitute a significant advance in the field of obstetrics. A major obstacle against developing new strategies for diagnosing and predicting PE is our relatively limited understanding of the disease. For example, inadequate placental perfusion, hypoxia, and/or ischemia are all believed to play a role in the placental release of circulating soluble factors that can have widespread consequences, including maternal systemic endothelial damage and dysfunction, vasoconstriction, and end-organ ischemia, ultimately resulting in the clinical symptoms associated with PE [15, 28, 29]. The important role of widespread endothelial dysfunction in PE is underscored by the finding that applying serum obtained from preeclamptic women to in vitro cultured endothelial cells causes cytotoxicity [17, 30].

We previously studied the role of CYFRA 21–1 in PE and found significantly higher levels of CYFRA 21–1 in the serum of 32 preeclamptic women compared to 32 matched controls [20]. Our current results, which are based on a larger number of cases, support this previous finding. Here, we found that the serum levels of CYFRA 21–1 are approximately twofold higher in women with PE compared to women with an uneventful pregnancy. Intrigued by these findings, we hypothesized that the increased levels of CYFRA 21–1 in women with PE reflect endothelial damage and/or dysfunction due to circulating factors of placental origin. Given that the pathogenic changes responsible for the endothelial dysfunction clearly precede the resulting clinical symptoms associated with PE, we further hypothesized that changes in the serum concentration of CYFRA 21–1 also precede the clinical symptoms of PE. Interestingly, our analysis revealed lower levels of serum CYFRA 21–1 early in pregnancy (i.e., at visits 1 and 2, corresponding to gestational week 11–13 and 14–17, respectively) in women who subsequently developed PE compared to women whose pregnancy remained uneventful. Nevertheless, the dynamics of maternal serum CYFRA 21–1 levels differed between the asymptomatic women who later developed PE and the women who remained asymptomatic. For example, CYFRA 21–1 levels were higher in the PE_long group at visits 3 through 6; however, these differences were not statistically significant.

As an influence of maternal BMI and age on CYFRA 21–1 levels cannot be ruled out, we decided to calculate another mixed model, which additionally included the maternal variables pre-pregnancy BMI and age as predictors. The results of this analysis suggest a link between maternal BMI and maternal age, and levels of CYFRA 21–1 independently of future PE risk. The reasons underlying this link are unclear. One could hypothesize that maternal obesity is associated with endothelial dysfunction, abnormalities in placental angiogenesis, or both. Interestingly, our adjusted model revealed that the effect of elevated BMI led to a slight reduction of CYFRA 21–1 levels, which might be explained by an increased volume of distribution in women with elevated BMI. This issue, more specifically the effect of maternal BMI on serum angiogenic markers in pregnancy, was already adressed by other groups [31, 32]. In a longitudinal study, Zera et al. found associations between maternal BMI, sFlt-1, and PlGF in pregnancies affected by placental dysfunction and in normal pregnancies [33]. As the exact mechanisms underlying the association between maternal obesity and ischemic placental diseases like PE are still a matter of speculation, (i) the association of maternal characteristics like BMI and age with placental dysfunction and subsequent endothelial damage, (ii) the increased volume of distribution in obese women, and (iii) the influence of these characteristics on serum levels of biomarkers for PE deserve increased attention in future studies.

We also investigated the value of using maternal CYFRA 21–1 to predict the risk of developing PE later in pregnancy. With respect to the long-term predictive power of CYFRA 21–1, serum samples collected up until gestational week 22 (i.e., visits 1, 2, and 3) revealed no predictive power. Next, we analyzed samples obtained at later gestational periods (i.e., visits 4, 5, and 6) in order to evaluate the short-term predictive power of CYFRA 21–1. This analysis revealed that CYFRA 21–1 to some extent has short-term predictive power, as the AUC of the ROC curves for the increase in CYFRA 21–1 between two subsequent visits increased incrementally with increasing gestational age. We speculate that these findings may reflect the increasing degree of endothelial damage that occurs until the onset of visible symptoms associated with PE. Therefore, CYFRA 21–1 is an interesting candidate molecule for future studies investigating promising biomarkers for predicting the short-term occurrence of PE.

We acknowledge that the relatively limited number of patients in our study should be considered when interpreting the results. In particular, the small size of the PE_long group (i.e., ten patients) yielded relatively wide confidence intervals for the AUCs, thereby precluding our ability to draw definitive conclusions regarding the power of CYFRA 21–1 in predicting the subsequent onset of PE. However, given the low prevalence of PE in industrialized countries, it is extremely difficult to obtain large numbers of PE cases using a prospective longitudinal study design. Thus, in our study, only ten out of 461 patients who were initially recruited to the longitudinal arm of our Biobank ultimately met the criteria for being assigned to the PE_long group. Therefore, we included an additional study group comprised of symptomatic women with PE, thereby increasing the strength of our study.

Another potential limitation of our study is the relatively low number of samples obtained from PE cases at visit 7 (i.e., after gestational week 37). In clinical practice, the routine standard of care is to induce labor—or if warranted, deliver by cesarean section—in symptomatic women who present with either confirmed or suspected PE at gestation week 37 or later. Therefore, even our PE_state group (which contained 50 cases) included only a few samples from women with generally mild cases of PE at visit 7. This low number of samples late in gestation may explain at least part of the lack of difference in CYFRA 21–1 levels between preeclamptic women and control cases at term.

On the other hand, the prospective longitudinal design of the Biobank and the strictly predefined endpoints resulted in a well-characterized patient cohort that was monitored closely throughout the entire pregnancy. This robust study design enabled us to accurately measure the dynamics of maternal CYFRA 21–1 throughout pregnancy. In addition, because CYFRA 21–1 is also an established marker for various types of cancer, the reference values for CYFRA 21–1 that we obtained may help validate CYFRA 21–1 as a tumor marker in pregnant women.

## Conclusion

Increased levels of serum CYFRA 21–1 in preeclamptic women may reflect endothelial damage and dysfunction due to circulating factors of placental origin. By comparing women whose pregnancy was complicated by PE with women whose pregnancy was uneventful, we found that CYFRA 21–1 is a promising biomarker for diagnosing PE. Although CYFRA 21–1 seems to have limited value for predicting the long-term occurrence of preeclampsia, our findings indicate that this marker may be useful for predicting the short-term occurrence of preeclampsia in asymptomatic women. Future prospective longitudinal studies will help determine whether this relatively simple, non-invasive test can be used to diagnose and/or predict preeclampsia.

## Abbreviations

95 % CI: 95 % confidence interval
ANOVA: Analysis of variance
AUC: Area under the curve
BMI: Body mass index
CYFRA 21–1: Cytokeratin fragment 21–1
GA: Gestational age
NICU: Neonatal intensive care unit
PE: Preeclampsia
PlGF: Placental growth factor
ROC: Receiver operating characteristics
SE: Standard error
sFlt-1: soluble fms-like tyrosine kinase 1

## References

[1] Steegers EA, von Dadelszen P, Duvekot JJ, Pijnenborg R (2010). Pre-eclampsia. Lancet.

[2] Waterstone M, Bewley S, Wolfe C (2001). Incidence and predictors of severe obstetric morbidity: case–control study. BMJ.

[3] Hahn S, Huppertz B, Holzgreve W (2005). Fetal cells and cell free fetal nucleic acids in maternal blood: new tools to study abnormal placentation?. Placenta.

[4] Myatt L (2002). Role of placenta in preeclampsia. Endocrine.

[5] Schweizer J, Bowden PE, Coulombe PA, Langbein L, Lane EB, Magin TM, Maltais L, Omary MB, Parry DA, Rogers MA, Wright MW (2006). New consensus nomenclature for mammalian keratins. J Cell Biol.

[6] Allard WJ, Matera J, Miller MC, Repollet M, Connelly MC, Rao C, Tibbe AG, Uhr JW, Terstappen LW (2004). Tumor cells circulate in the peripheral blood of all major carcinomas but not in healthy subjects or patients with nonmalignant diseases. Clin Cancer Res.

[7] Schmidt M, Hoffmann B, Beelen D, Gellhaus A, Winterhager E, Kimmig R, Kasimir-Bauer S (2008). Detection of circulating trophoblast particles in peripheral maternal blood in preeclampsia complicated pregnancies. Hypertens Pregnancy.

[8] Okamura K, Takayama K, Izumi M, Harada T, Furuyama K, Nakanishi Y (2013). Diagnostic value of CEA and CYFRA 21–1 tumor markers in primary lung cancer. Lung Cancer.

[9] Inaba N, Negishi Y, Fukasawa I, Okajima Y, Ota Y, Tanaka K, Matsui H, Iwasaki H, Sudo H, Tanaka N (1995). Cytokeratin fragment 21–1 in gynecologic malignancy: comparison with cancer antigen 125 and squamous cell carcinoma-related antigen. Tumour Biol.

[10] Brockmann JG, St Nottberg H, Glodny B, Heinecke A, Senninger NJ (2000). CYFRA 21–1 serum analysis in patients with esophageal cancer. Clin Cancer Res.

[11] Sharma SK, Bhat S, Chandel V, Sharma M, Sharma P, Gupta S, Sharma S, Bhat AA (2015). Diagnostic utility of serum and pleural fluid carcinoembryonic antigen, and cytokeratin 19 fragments in patients with effusion from nonsmall cell lung cancer. J Carcinog.

[12] Yang DW, Zhang Y, Hong QY, Hu J, Li C, Pan BS, Wang Q, Ding FH, Ou JX, Liu FL (2015). Role of a serum-based biomarker panel in the early diagnosis of lung cancer for a cohort of high-risk patients. Cancer.

[13] Cui C, Sun X, Zhang J, Han D, Gu J (2014). The value of serum Cyfra21-1 as a biomarker in the diagnosis of patients with non-small cell lung cancer: a meta-analysis. J Cancer Res Ther.

[14] Longtine MS, Chen B, Odibo AO, Zhong Y, Nelson DM (2012). Villous trophoblast apoptosis is elevated and restricted to cytotrophoblasts in pregnancies complicated by preeclampsia, IUGR, or preeclampsia with IUGR. Placenta.

[15] Huppertz B, Kadyrov M, Kingdom JC (2006). Apoptosis and its role in the trophoblast. Am J Obstet Gynecol.

[16] Musci TJ, Roberts JM, Rodgers GM, Taylor RN (1988). Mitogenic activity is increased in the sera of preeclamptic women before delivery. Am J Obstet Gynecol.

[17] Roberts JM, Taylor RN, Musci TJ, Rodgers GM, Hubel CA, McLaughlin MK (1989). Preeclampsia: an endothelial cell disorder. Am J Obstet Gynecol.

[18] Schrocksnadel H, Daxenbichler G, Artner E, Steckel-Berger G, Dapunt O (1992). Tissue polypeptide antigen and pre-eclampsia. Lancet.

[19] Tempfer CB, Bancher-Todesca D, Zeisler H, Schatten C, Husslein P, Gregg AR (2000). Placental expression and serum concentrations of cytokeratin 19 in preeclampsia. Obstet Gynecol.

[20] Kuessel L, Wild J, Haslacher H, Perkmann T, Ristl R, Zeisler H, Schmid M (2014). Urine and serum concentrations of Cytokeratin 19 in preeclampsia. Eur J Obstet Gynecol Reprod Biol.

[21] Li XL, Dong X, Xue Y, Li CF, Gou WL, Chen Q (2014). Increased expression levels of E-cadherin, cytokeratin 18 and 19 observed in preeclampsia were not correlated with disease severity. Placenta.

[22] Bulletins--Obstetrics ACoP: ACOG practice bulletin (2002). Diagnosis and management of preeclampsia and eclampsia. Number 33, January 2002. Obstet Gynecol.

[23] von Dadelszen P, Magee LA, Roberts JM (2003). Subclassification of preeclampsia. Hypertens Pregnancy.

[24] Duley L (1992). Maternal mortality associated with hypertensive disorders of pregnancy in Africa, Asia, Latin America and the Caribbean. Br J Obstet Gynaecol.

[25] Verlohren S, Galindo A, Schlembach D, Zeisler H, Herraiz I, Moertl MG, Pape J, Dudenhausen JW, Denk B, Stepan H (2010). An automated method for the determination of the sFlt-1/PIGF ratio in the assessment of preeclampsia. Am J Obstet Gynecol.

[26] Verlohren S, Herraiz I, Lapaire O, Schlembach D, Zeisler H, Calda P, Sabria J, Markfeld-Erol F, Galindo A, Schoofs K (2014). New gestational phase-specific cutoff values for the use of the soluble fms-like tyrosine kinase-1/placental growth factor ratio as a diagnostic test for preeclampsia. Hypertension.

[27] Zeisler H, Llurba E, Chantraine F, Vatish M, Staff AC, Sennstrom M, Olovsson M, Brennecke SP, Stepan H, Allegranza D (2016). Predictive Value of the sFlt-1:PlGF Ratio in Women with Suspected Preeclampsia. N Engl J Med.

[28] Maynard SE, Karumanchi SA (2011). Angiogenic factors and preeclampsia. Semin Nephrol.

[29] Redman CW, Sargent IL (2010). Immunology of pre-eclampsia. Am J Reprod Immunol.

[30] Rodgers GM, Taylor RN, Roberts JM (1988). Preeclampsia is associated with a serum factor cytotoxic to human endothelial cells. Am J Obstet Gynecol.

[31] Masuyama H, Segawa T, Sumida Y, Masumoto A, Inoue S, Akahori Y, Hiramatsu Y (2010). Different profiles of circulating angiogenic factors and adipocytokines between early- and late-onset pre-eclampsia. BJOG.

[32] Suwaki N, Masuyama H, Nakatsukasa H, Masumoto A, Sumida Y, Takamoto N, Hiramatrsu Y (2006). Hypoadiponectinemia and circulating angiogenic factors in overweight patients complicated with pre-eclampsia. Am J Obstet Gynecol.

[33] Zera CA, Seely EW, Wilkins-Haug LE, Lim KH, Parry SI, McElrath TF (2014). The association of body mass index with serum angiogenic markers in normal and abnormal pregnancies. Am J Obstet Gynecol.

